# Ethnic inequalities in multiple comorbidities among people with psychosis

**DOI:** 10.1192/j.eurpsy.2022.864

**Published:** 2022-09-01

**Authors:** D. Fonseca De Freitas, M. Khondoker, J. Nazroo, R. Hayes, K. Bhui

**Affiliations:** 1 Institute of Psychiatry, Psychology & Neuroscience, King’s College London, Psychological Medicine, London, United Kingdom; 2 University of East Anglia, Norwich Medical School, Norwich, United Kingdom; 3 University of Manchester, School Of Social Sciences, Manchester, United Kingdom; 4 Institute of Psychiatry, Psychology, and Neuroscience, King’s College London, Department Of Psychological Medicine, London, United Kingdom; 5 Universiy of Oxford, Dept Of Psychiatry And Nuffield Dept Of Primary Care Health Sciences, Oxford, United Kingdom

**Keywords:** multimorbidity, Psychosis, health inequalities, ethnicity

## Abstract

**Introduction:**

Studies have shown ethnic inequalities in health, with a higher incidence of illnesses among people of some minoritised ethnic groups. Furthermore, it has been observed that people with severe mental illnesses have a higher risk for multimorbidity. However, no study has investigated ethnic disparities in comorbidity in people with a schizophrenia spectrum disorder.

**Objectives:**

This study investigates potential ethnic disparities in physical health comorbidity in a cohort of people with psychosis.

**Methods:**

Using a cross-sectional design, we identified service-users of the South London and Maudsley NHS Trust who were diagnosed with a schizophrenia spectrum disorder between 2007 and 2020. We assessed the prevalence of asthma, bronchitis, diabetes, hypertension, low blood pressure, overweight or obesity, and rheumatoid arthritis. Latent class analyses were used to investigate distinct profiles of comorbidity. Multinomial regression was then used to investigate ethnic disparities in these profiles. The regression model was adjusted for gender, age, neighbourhood deprivation, smoking and duration of care.

**Results:**

On a sample of 23,418 service-users with psychosis, we identified two classes of comorbidity: low comorbidity and multiple comorbidities. Compared to the White British ethnicity, a higher risk for multiple comorbidities was observed for people with any Black background, Indian, Pakistani, Asian British, and mixed-race ethnicities. Furthermore, Black African women had a significantly higher risk for multiple comorbidities than their male counterparts.

**Conclusions:**

Ethnic disparities are observed in multiple comorbidities among people with psychosis. Further research is needed to understand the impact of these disparities, especially in relation to mortality.

**Disclosure:**

No significant relationships.

